# Autosomal recessive spastic ataxia of Charlevoix Saguenay (ARSACS): expanding the genetic, clinical and imaging spectrum

**DOI:** 10.1186/1750-1172-8-41

**Published:** 2013-03-15

**Authors:** Matthis Synofzik, Anne S Soehn, Janina Gburek-Augustat, Julia Schicks, Kathrin N Karle, Rebecca Schüle, Tobias B Haack, Martin Schöning, Saskia Biskup, Sabine Rudnik-Schöneborn, Jan Senderek, Karl-Titus Hoffmann, Patrick MacLeod, Johannes Schwarz, Benjamin Bender, Stefan Krüger, Friedmar Kreuz, Peter Bauer, Ludger Schöls

**Affiliations:** 1 Department of Neurodegenerative Diseases, Hertie-Institute for Clinical Brain Research, University of Tübingen, Hoppe-Seyler-Str. 3, Tübingen, 72076, Germany; 2 German Research Center for Neurodegenerative Diseases (DZNE), Tübingen, Germany; 3 Institute of Medical Genetics and Applied Genomics, University of Tübingen, Tübingen, Germany; 4 Department of Neuropediatrics, University of Tübingen, Tübingen, Germany; 5 Institute of Human Genetics, Technische Universität München, Munich, Germany; 6 CeGaT GmbH, Center for Genomics and Transcriptomics, Tübingen, Germany; 7 Institute of Human Genetics, RWTH Aachen University, Aachen, Germany; 8 Department of Neurology, Friedrich-Baur Institute, Ludwig-Maximilian University Munich, Munich, Germany; 9 Department of Neuroradiology, University of Leipzig, Leipzig, Germany; 10 Medical Genetics Clinic, Victoria General Hospital, Victoria, Canada; 11 Hospital Mühldorf am Inn, Mühldorf, Germany; 12 Dept. of Neuroradiology, University of Tübingen, Tübingen, Germany; 13 Gemeinschaftspraxis für Humangenetik, Dresden, Germany

**Keywords:** Ataxia, Recessive ataxia, Spastic ataxia, Early onset ataxia, Spasticity, Genetics, Magnetic resonance imaging, Electrophysiology, Thin corpus callosum, Charcot Marie Tooth, Hereditary spastic paraplegia

## Abstract

**Background:**

Mutations in *SACS*, leading to autosomal-recessive spastic ataxia of Charlevoix-Saguenay (ARSACS), have been identified as a frequent cause of recessive early-onset ataxia around the world. Here we aimed to enlarge the spectrum of *SACS* mutations outside Quebec, to establish the pathogenicity of novel variants, and to expand the clinical and imaging phenotype.

**Methods:**

Sequencing of *SACS* in 22 patients with unexplained early-onset ataxia, assessment of novel *SACS* variants in 3.500 European control chromosomes and extensive phenotypic investigations of all *SACS* carriers.

**Results:**

We identified 11 index patients harbouring 17 novel *SACS* variants. 9/11 patients harboured two variants of at least probable pathogenicity which were not observed in controls and, in case of missense mutations, were located in highly conserved domains. These 9 patients accounted for at least 11% (9/83) in our series of unexplained early onset ataxia subjects. While most patients (7/9) showed the classical ARSACS triad, the presenting phenotype reached from pure neuropathy (leading to the initial diagnosis of Charcot-Marie-Tooth disease) in one subject to the absence of any signs of neuropathy in another. In contrast to its name “spastic ataxia”, neither spasticity (absent in 2/9=22%) nor extensor plantar response (absent in 3/9=33%) nor cerebellar ataxia (absent in 1/9=11%) were obligate features. Autonomic features included urine urge incontinence and erectile dysfunction. Apart from the well-established MRI finding of pontine *hypo*intensities, all patients (100%) showed *hyper*intensities of the lateral pons merging into the (thickened) middle cerebellar peduncles. In addition, 63% exhibited bilateral parietal cerebral atrophy, and 63% a short circumscribed thinning of the posterior midbody of the corpus callosum. In 2 further patients with differences in important clinical features, VUS class 3 variants (c.1373C>T [p.Thr458Ile] and c.2983 G>T [p.Val995Phe]) were identified. These variants were, however, also observed in controls, thus questioning their pathogenic relevance.

**Conclusions:**

We here demonstrate that each feature of the classical ARSACS triad (cerebellar ataxia, spasticity and peripheral neuropathy) might be missing in ARSACS. Nevertheless, characteristic MRI features – which also extend to supratentorial regions and involve the cerebral cortex – will help to establish the diagnosis in most cases.

## Background

Autosomal-recessive spastic ataxia of Charlevoix-Saguenay (ARSACS) was for long regarded as a subtype of early onset ataxia restricted to the Quebec region due to founder mutations from a French ancestor. After cloning of the responsible gene, *SACS*, it became evident that ARSACS is not limited to Quebec, and more than 100 different pathogenic mutations have now been identified worldwide [1]. Most ARSACS patients show a typical triad of early-onset cerebellar ataxia, lower limb spasticity and peripheral neuropathy. Together with characteristic T2-hypointensities in the pons [2,3], these phenotypic features help to identify ARSACS in patients with unexplained ataxia. However, three mutation-proven individuals with an unusual phenotype (lacking either spasticity or peripheral neuropathy) have been described so far [1,4,5], indicating that the classical triad might not be the presenting feature in *all* ARSACS patients, and that ARSACS might be underdiagnosed in patients with atypical phenotypes. Moreover, the presumed high frequency of allele carriers around the world [6] suggests that many subjects will be identified in the future with *SACS* variants of unknown significance, thus warranting the identification of further phenotypic features that help to establish the clinical diagnosis.

Here we aimed (i) to expand the spectrum of *SACS* mutations outside Quebec and to establish the pathogenicity of novel variants, (ii) to identify phenotypic characteristics that are shared by most ARSACS patients and that further help to identify ARSACS in patients with unexplained ataxia, spasticity or neuropathy, and (iii) to assess non-classical presenting phenotypes that indicate additional disease groups, in which mutational screening of *SACS* should be considered in the molecular genetic work-up.

## Methods

From 2005 until 2012 we recruited a consecutive series of 172 index patients in the ataxia outpatient clinics of the Departments of Neurology and Pediatric Neurology at the University Clinic in Tübingen (Germany) with (i) progressive degenerative ataxia with age of onset ≤30 years, (ii) absence of ataxia in the parental generation, and (iii) genetic exclusion of common subtypes of dominant ataxias (SCA 1, 2, 3, 6, 7, and 17). Mutations causing Friedreich-, *POLG*- and *C10orf2* (*PEO1/Twinkle*)-ataxia were screened in the whole cohort. A molecular diagnosis could not be established in 83/172 patients (48%). 22 of these 83 patients with unexplained ataxia were screened for ARSACS by direct sequencing of the *SACS* gene (for details, see Additional file 1). These 22 patients were selected for direct sequencing by 2 criteria: (1) pyramidal tract signs to the lower extremity in addition to the degenerative ataxia (20/22 patients); or (2) variant abnormalities including the *SACS* region detected by preceding homozygosity mapping and/or whole-exome sequencing approaches (2/22 patients). In all index patients with only one heterozygous or apparently homozygous *SACS* variants, macro-deletions were excluded by MLPA (see Additional file 1 for details). Following earlier recommendations on variant classification and reporting [7], the likelihood of pathogenicity of novel sequence variants was graded according to a 5-class-system: class 1 = not pathogenic; class 2 = variant of uncertain clinical significance (VUS), likely not pathogenic; class 3 = VUS, uncertain pathogenicity; class 4 = VUS, likely pathogenic; class 5 = definitely pathogenic [7]. Only patients harbouring two variants of class 3 or higher are reported here (see Additional file 1 for details on variant classes and assignment of variants). The frequency of the identified *SACS* variants was determined in 3.500 European control chromosomes from individuals with unrelated phenotypes analyzed by exome sequencing (for methodological details, see [8]) in Munich, Germany. In addition, the frequency was assessed in the exome variant server (EVS) of the National Heart, Lung, and Blood Institute GO Exome Sequencing Project (Seattle, WA, USA; URL: evs.gs.washington.edu/EVS/) with the corresponding nucleotide positions being analyzed in >8.000 European and >4.000 African American alleles and in the Database of Single Nucleotide Polymorphisms (dbSNP; Bethesda: National Center for Biotechnology Information, National Library of Medicine [dbSNP Build ID: 137]; available from: http://www.ncbi.nlm.nih.gov/SNP/). *In-silico* predictions of the pathogenicity of point mutations were performed using PolyPhen-2 [9], SIFT [10] and MutationTaster [11]. Cosegregation of novel variants was confirmed in all families with a second affected family member. Compound heterozygosity was confirmed by segregation analysis where parental DNA was available.

Each mutation carrier was clinically characterized by a movement disorder specialist and investigated by nerve conduction studies (NCS), motor evoked potentials (MEP), standard funduscopy, and routine magnetic resonance imaging (MRI) including T1-, T2-, diffusion-weighted images (DWI) and fluid attenuated inversion recovery T2 (FLAIR) images. The presence vs. absence of thinning of the corpus callosum and of atrophy of the cerebellar and parietal cortex were evaluated independently by two examiners (M.S.; B.B.), and the inter-rater reliability was determined by Cohen’s kappa coefficient. Its value was interpreted following Landis and Koch [12]. In case of disagreement, the more conservative assessment (i.e. the absence of thinning and atrophy, respectively) was adopted for final judgement.

## Results

### Genetic spectrum

**Figure 1:**
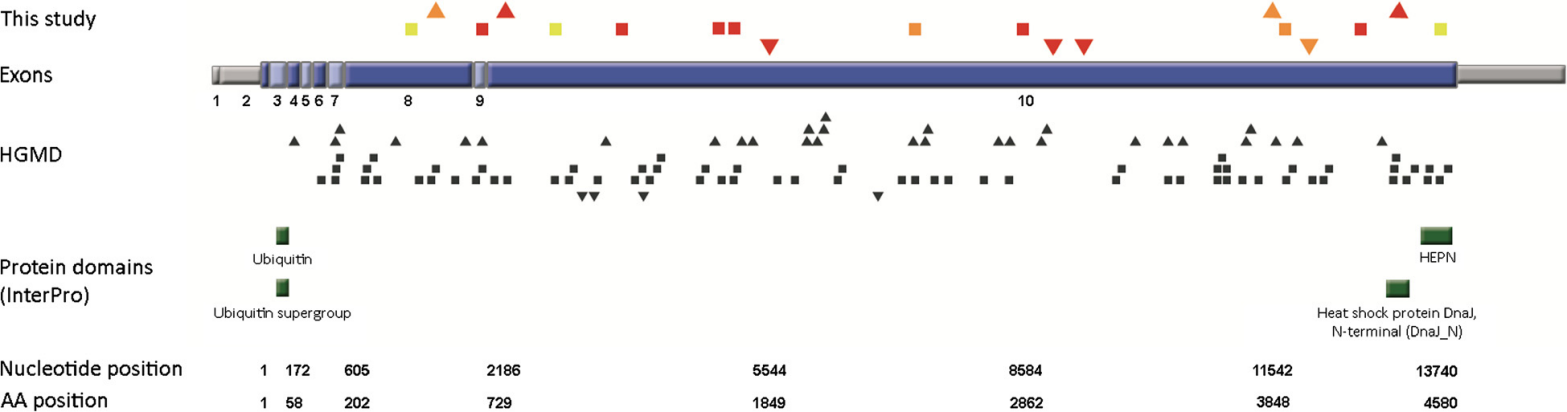
***SACS *mutations.** Graphical overview of mutations found in this and other studies (Human Gene Mutation database, except gross deletions). Different mutation types are marked with different symbols: missense mutations = squares; insertions/duplications = triangles with downward orientation; deletions = triangles with upward orientation. Sequence variations identified in this study are coloured, with the colour indicating the pathogenicity class: red = pathogenic, orange = likely pathogenic, yellow = uncertain. Known protein domains of the sacsin protein are highlighted in green. AA = amino acid; HGMD = Human Gene Mutation Database.

In total, 19 different *SACS* variants were identified across different exons (Figure 1). 17 out of 19 variants are novel, 2 have already been identified earlier (p. Arg728*; p.Gln1709*; [6]) (see Table 1). The identified variants comprised 6 nonsense mutations, 5 missense mutations, 5 frameshift mutations, 5 in-frame deletions, and 1 in-frame duplication. These variants were observed in 11 index patients. In three families with known parental consanguinity, homozygous *SACS* variants were observed (Table 1). Four index patients had a positive family history with affected siblings. Cosegregation of the respective variants was demonstrated in all of them. No macro-deletion or duplications were identified by MLPA.

**Table 1:** ***SACS *mutations**

**Family No.**	**Nucleotide change**^**1**^	**Amino acid change**	**Mutation type**	**Exon**	**Allele frequency in 1846 controls**	**dbSNP**	**EVS**	**PhyloP**^**2 **^**[-14,1;6,4]**	**Grantham distance**^**3 **^**[0–215]**	**In-silico predictions**^**4**^	**Novel variant**	**Pathogenicity class**^**5**^
**Patients with 2 pathogenic or likely pathogenic *SACS *variants**												
**#1 index**	c.9305_9306insT	p.Leu3102Phefs*8	frame shift	10	0	-	-	NA	NA	NA	yes	**Pathogenic (class 5)**
	c.9305_9306insT	p.Leu3102Phefs*8										
#1 sister	see above											
**#2**	c.2182C>T	p.Arg728*	nonsense	9	0	-	-	ND	NA	NA	no^6^	**Pathogenic (class 5)**
	c.13056delT	p.Phe4352Leufs*11	frame shift	10	0	-	-	NA	NA	NA	yes	**Pathogenic (class 5)**
**#3 index**	c.3769G>T	p.Gly1257*	nonsense	10	0	-	-	ND	NA	NA	yes	**Pathogenic (class 5)**
	c. 8584A>T	p.Lys2862*	nonsense	10	0	-	-	ND	NA	NA	yes	**Pathogenic (class 5)**
#3 sister	see above											
**#4 index**	c.11542_11544del	p.Ile3848del	in-frame deletion	10	0	-	-	NA	NA	NA	yes	**Likely pathogenic (VUS class 4)**
	c.11542_11544del	p.Ile3848del										
#4 brother	see above											
**#5 index**	c.2387delT	p.Leu796Tyrfs*13	frame shift	10	0	-	-	NA	NA	NA	yes	**Pathogenic (class 5)**
	c.11984_11986 dupTGT	p.L3995dup	in-frame duplication	10	0	-	-	NA	NA	NA	yes	**Likely pathogenic (VUS class 4)**
#5 sister	see above											
#6	c.11624 G>A	p.Arg3875His	missense	10	0	-	-	6.34	29	Deleterious	yes	**Likely pathogenic (VUS class 4)**
	c.1647_1658del	p.Leu549_Leu552del	In-frame deletion	8	0	-	-	NA	NA	NA	yes	**Likely pathogenic (VUS class 4)**
	c.13538 G>A	p.Ser4513Asn	missense	10	0	rs138328181 (0% ESP Cohort pop)	0.02% (EA), 0% (AA)	3.43	46	Contradictory	yes	**Uncertain (VUS class 3)**
#7	c.5544dupA	p.Val1849Serfs*48	frame shift	10	0	-	-	NA	NA	NA	yes	**Pathogenic (class 5)**
	c.12603C>A	p.Tyr4201*	nonsense	10	0	-	-	ND	NA	NA	yes	**Pathogenic (class 5)**
#8	c.7277 G>C	p.Arg2426Pro	missense	10	0	-	-	5.69	103	Deleterious	yes	**Likely pathogenic (VUS class 4)**
	c.8920_8923dup	p.Tyr2975Phefs*29	frame shift	10	0	-	-	NA	NA	NA	yes	**Pathogenic (class 5)**
#9	c.4954C>T	p.Gln1652*	nonsense	10	0	-	-	ND	NA	NA	yes	**Pathogenic (class 5)**
	c.5125C>T	p.Gln1709*	nonsense	10	0	-	-	ND	NA	NA	no^6^	**Pathogenic (class 5)**
**Patients with 2 *SACS *variants of uncertain clinical significance class 3 (VUS3)**												
#10	c.1373C>T	p.Thr458Ile	missense	8	14/3500	rs61729954 (0.3% ESP Cohort pop; 3.1% AGI ASP pop)	0.33% (EA); 0.05% (AA)	6.26	89	Deleterious	yes	**Uncertain (VUS class 3)**
	c.1373C>T	p.Thr458Ile										
#11	c.1373C>T (see patient #10)	p.Thr458Ile	missense	8	14/3500	rs61729954 (0.3% ESP Cohort pop; 3.1% AGI ASP pop)	0.33% (EA); 0.05% (AA)	6.26	89	Deleterious	yes	**Uncertain (VUS class 3)**
	c.2983 G>T	p.Val995Phe	missense	10	11/3500	rs142967124 (0.1% ESP Cohort pop)	0.24% (EA); 0.02% (AA)	-0.2	50	Contradictory	yes	**Uncertain (VUS class 3)**

*EA*= European alleles; *AA*= African American alleles; *NA* = not applicable; *ND* = not determined; rs = reference single nucleotide polymorphism (SNP) identifier; *VUS* = variant of uncertain clinical significance. ^1^ Nomenclature of sequence variants follows the recommendations by the Human Genome Variation Society (HGVS, http://www.hgvs.org/mutnomen/) and refers to the cDNA sequence NM_014363.4. ^2^ PhyloP was used as a conservation score as originally implemented by Pollard and colleagues. It allows the rating of nucleotides from “not conserved” (-14.1) to “highly conserved” (6.4) [13]. ^3^ The Grantham distance was used to evaluate physico-chemical differences of amino acids (0 = no physico-chemical differences; 215 = large differences) [14]. ^4^ In-silico predictions were performed using PolyPhen-2 [9], SIFT [10] and MutationTaster [11]^5^ Classification of novel sequence variants is according to [7]. ^6^ Reported in [6].

Fourteen of the novel 17 variants were not observed in controls and in the EVS (Table 1) and - in case of missense mutations -were evolutionary highly conserved (for conservation score PhyloP, see Table 1; for alignment data, see Additional file 2, each only for missense mutations). Two *SACS* variants - p.Thr458Ile and p.Val995Phe - were also observed in heterozygous state in 3.500 control chromosomes (p.Thr458Ile: 14/3.500=0.4%; p.Val995Phe: 11/3.500=0.3%), in dbSNP (p.Thr458Ile: 0.3% ESP Cohort pop; rs61729954; p.Val995Phe: 0.1% ESP Cohort pop; rs142967124) and in the exome variant server (p.Thr458Ile: 0.33% European alleles; p.Val995Phe: 0.24% European alleles) (see Table 1). The p.Thr458Ile variant was predicted to be deleterious by three different *in silico* software tools (PolyPhen-2, SIFT, MutationTaster). It leads to a change of an evolutionary highly conserved amino acid (PhyloP Score: 6.26) (Table 1 and Additional file 2). However, the physico-chemical differences of the involved amino acids are only moderate. This finding together with their occurrence in the general population leads to the classification as VUS class 3 (uncertain pathogenicity). The p.Val995Phe variant is predicted to be deleterious only by SIFT, and affects an evolutionary less conserved amino acid (PhyloP Score: -0.2) (Table 1 and Additional file 2), thus also leading to the classification as VUS class 3. Due to the frequent observation of these two variants in controls and their uncertain significance, the two patients harbouring either one of these variants (patient #10: p.[Thr458Ile];[Thr458Ile]) or the combination of them (patient #11: p.[Thr458Ile]; [Val995Phe]) were analyzed separately from the other 9 patients with two SACS variants of ≥ class 4.

One SACS variant – c.13538 G>A (p.Ser4513Asn) - was not observed in controls, but was found in the EVS (0.02%) and was only moderately conserved. This variant was found in a patient with two additional *SACS* variants (patient #6), namely c.11624 G>A (p.Arg3875His) and c.1647_1658del (p. Leu549_Leu552del). Only these two variants, but not the first variant, were considered to be likely pathogenic (VUS class 4), since they were absent in controls and the EVS and were highly evolutionary conserved. Unfortunately, DNA of the parents was not available for testing allele segregation.

In one index patient (patient #2), an autosomal-dominant ataxia had initially been assumed, based on the positive family history for a three-generation late-onset (>30 years), slowly progressive, pure cerebellar autosomal-dominant ataxia in her paternal relatives (in a aunt, cousin, and grandfather; or pedigree see Additional file 3). However, the father of the index patient did not show any ataxia (examined at age 80 years by M.S.) and the index patient herself showed a strikingly different early-onset ataxia. To rule out a possible digenic ataxia, also the following SCAs were excluded in the index patient: SCA 8,10, 11, 14, 15, 27 and DRPLA. The affected paternal aunt and cousin did not show any of the two (clearly pathogenic) *SACS* variants that were observed in the index patient. This illustrates that two different hereditary ataxias should be considered in pedigrees where the ataxia features differ strongly between pedigree members, e.g. early onset vs. late onset ataxia or multisystemic vs. pure cerebellar degeneration.

### Phenotypic spectrum of index patients with two ARSACS variants of at least probable pathogenicity

78% (7/9) of the 9 index patients with at least two variants of VUS class 4 (likely pathogenic [7]) or pathogenic mutations showed the typical ARSACS phenotype of typical early-onset triad of cerebellar ataxia, lower limb pyramidal tract signs (spasticity and extensor plantar response in 6/7; only spasticity in 1/7 [patient #9]) and peripheral neuropathy, which was of axonal-demyelinating sensorimotor type in all (7/7) subjects (Table 2). 1/9 patients (patient #4) showed no spasticity, extensor plantar response or cerebellar ataxia, but marked axonal-demyelinating sensorimotor neuropathy (leading to mild subclinical afferent ataxia). The same was true for his likewise affected brother. In turn, in another patient (patient #8), cerebellar ataxia was present, but electrophysiological evidence of peripheral neuropathy absent. Neither did this patient show spasticity or an extensor plantar response. Thus, neither spasticity (absent in 2/9=22%) nor extensor plantar response (absent in 3/9=33%) nor cerebellar ataxia (absent in 1/9=11%) are obligate features in ARSACS.

**Table 2:** **Clinical characteristics of patients with *SACS *variants**

**Family No.**	**SACS variant**	**Ethnical background**	**Current age (y)/sex**	**Age at onset (y)**	**First clinical sign**	**Current SARA**	**Cerebellar ataxia**	**Areflexia muscle tendon reflexes**	**Pyramidal features**	**Autonomic signs**
**patients with 2 likely pathogenic *SACS *variants**										
**#1 index**	p.[Leu3102Phefs*8];[Leu3102Phefs*8]	Turkey	31/f	1	delayed motor development	12	Dysarthria, nystagmus, dysmetria UE	ATR	spasticity LE>UE; Bab +/+	urge incontinence urine
#1 sister	see above		30/f	11	gait disturbance	10	Nystagmus, dysmetria UE+LE	ATR	spasticity LE>UE; Bab +/+	urge incontinence urine
**#2**	p.[Arg728*];[Phe4352Leufs*11]	German	38/f	6	gait disturbance	22	nystagmus, dysmetria UE+LE	ATR	spasticity, Bab +/+	urge incontinence urine
**#3 index**	p.[Gly1257*]; [Lys2862*]	Greek	40/m	18	gait disturbance	22	nystagmus, dysarthria, dysmetria UE+LE	-	spasticity, Bab +/+	urge incontinence urine
#3 sister	see above		36/f	15	gait disturbance	n.d.	n.k.	n.k.	n.k.	urge incontinence urine
**#4 index**	p.[Ile3848del]; [Ile3848del]	Turkey	13/m	3	generalized muscle weakness, pes planus	2	-	-	none; Bab -/-	-
#4 brother	see above		6/m		pes planus	1	-	-	none; Bab -/-	-
**#5; index**	p.[Leu796Tyrfs*13];[L3995dup]	German	27/f	2	gait disturbance	25	nystagmus, dysmetria UE+LE	ATR	spasticity, Bab +/+	urge incontinence urine & faeces
#5, sister	see above		25/f	2	gait disturbance	n.a.	Dysarthria, dysmetria LE > UE	ATR	none, Bab -/-	urge incontinence urine
#6	p.[Arg3875His]; [Leu549_Leu552del]; [Ser4513Asn]	Macedonia	30/m	1	gait disturbance	9	dysmetria UE+LE -	ATR	spasticity, Bab+/+	-
#7	p.[Val1849Serfs*48]; [Tyr4201*]	German	50/f	28	gait disturbance	n.a.	nystagmus, dysarthria, dysmetria UE+ UE	ATR	spasticity; Bab +/+	urge incontinence
#8	p.[Arg2426Pro]; [Tyr2975Phefs*29]	German	6/m	2	gait disturbance	9	nystagmus, dysarthria, dysmetria UE+LE	-	none; Bab -/-	incontinence
#9	p.[Gln1652*]; [Gln1709*]	German	41/m	2	gait disturbance	n.a.	nystagmus, dysarthria, dysmetria UE+LE	ATR	spasticity; Bab -/-	urge incontinence, erectile dysfunction
**patients with 2 *SACS *variants of unknown significance**										
#10	p.[Thr458Ile]; [Thr458Ile]	Turkey	44/m	30	gait disturbance	8	nystagmus, slight dysmetria UE + LE, no dysarthria	-	none; Bab -/-	urge incontinence
#11	p.[Thr458Ile]; [Val995Phe]	German	43/m	20	gait ataxia	21	cerebellar oculomotor disturbance, dysarthria, dysphagia, intention tremor, dysmetria UE+LE	-	spasticity; Bab+/+	-

n.a.= not available; n.k., not known; y= years; m=male, f=female; ATR, Achilles Tendon Reflex; Bab=babinski sign right/left; SARA= Scale for the Assessment and Rating of Ataxia (according to [15]).

Urinary urge incontinence was present in 7/9 patients (77%), combined with faecal urge incontinence in 1 patient; erectile dysfunction was present in 1/3 male patients aged ≥16 years (33%) (Table 2). Standard funduscopy (available from 6/9 patients) revealed increased demarcation of retinal nerve fibres close to the optic disc in 1/6 (17%) patients (patient #5). No patient reported hearing loss (0%). MEPs (available from 8/9 patients) were abnormal to the legs in all patients (not evoked in 7/8, prolonged central motor conduction time in 1/8) and to the arms in 75% (prolonged central motor conduction time in all 6/8 patients), indicating that pyramidal damage does not only affect lower but also upper extremities in ARSACS (Table 3). This was true also for patient #4, in whom no clinical signs of pyramidal damage were observed, thus indicating that electrophysiology may reveal subclinical pyramidal damage in ARSACS.

**Table 3:** Electrophysiology and MRI findings

**family No.**	**SACS variants**	**Sensory nerve conduction sural or radial nerve**	**Motor nerve conduction tibial or ulnar nerve**	**Motor evoked potentials**	**Cerebellar atrophy**	**Arachnoid cyst posterior fossa**	**T2 hypo-intensities pons**	**T2 hyper-intensities lateral pons/MCP**	**Thickening of MCP**	**Bilateral parietal atrophy**	**Thin corpus callosum (MRI)**
**patients with 2 likely pathogenic *SACS *variants**											
#1	p.[Leu3102Phefs*8];[Leu3102Phefs*8]	Sur: no SNAP Rad: no SNAP	Tib:reduced CMAP+MNCV Uln: reduced MNCV	prolonged UE+LE	+, vermal	+	+	+	+	+	+
#2	p.[Arg728*];[Phe4352Leufs*11]	Sur: no SNAP Rad: no SNAP	Tib: reduced MNCV Uln: reduced MNCV	prologned UE, not evoked LE	+, pancerebellar	+	+	+	+	+	+
#3	p.[Gly1257*];[Lys2862*]	Sur: no SNAP Rad: no SNAP	Tib: no MSAP Uln: reduced MNCV	prolonged UE, not evoked LE	+, pancerebellar	+	+	+	+	+	+
#4	p.[Ile3848del];[Ile3848del]	Sur: no SNAP Rad: reduced CMAP+SNCV	Tib: reduced MNCV Uln: reduced MNCV	prolonged UE, not evoked LE	-, only asymmetry of cerebellar hemispheres	+	+	+	+	-	+
#5	p.[Leu796Tyrfs*13];[L3995dup]	Sur: no SNAP Rad: reduced CMAP+SNCV	Tib: reduced CMAP+MNCV Uln: reduced MNCV	prolonged UE, not evoked LE	+, vermal	+	+	+	+	-	+
#6	p.[Arg3875His]; [Leu549_Leu552del]; [Ser4513Asn]	Sur: reduced CMAP+SNCV Rad: reduced CMAP+SNCV	Tib: reduced CMAP+MNCV Uln: reduced MNCV	prolonged UE, not evoked LE	+, vermal	+	+	+	+	+	-
#7	p.[Val1849Serfs*48]; [Tyr4201*]	Sur: reduced CMAP+SNCV	Tib: reduced CMAP+MNCV	not evoked LE	+, pancerebellar	n.a.	n.a.	n.a.	n.a.	n.a.	n.a.
#8	p.[Arg2426Pro]; [Tyr2975Phefs*29]	Sur: normal	Tib: normal	not done	+, vermal	-	+	+	+	-	-
#9	p.[Gln1652*]; [Gln1709*]	Sur: no SNAP Rad: reduced SNAP+SNCV	Tib: reduced CMAP+MNCV Uln: reduced MNCV	not evoked LE	+, pancerebellar	-	+	+	+	+	-
**patients with 2 *SACS *variants of unknown significance**											
#10	p.[Thr458Ile]; [Thr458Ile]	Sur: normal	Tib: normal	normal UE+LE	slight vermal atrophy, herniation of tonsils	+	-	-	-	-	+
#11	p.[Thr458Ile]; [Val995Phe]	Sur: red SNAP Rad: red SNAP	Tib: red CMAP	prolonged UE+LE	+, pancerebellar	-	-	-	-	+	+

Sur=sural; rad= radial; Tib= tibial, Uln=ulnar; SNAP=sensory nerve action potential; SNCV=sensory nerve conduction velocity; CMAP=compound muscle action potential; MNCV=motor nerve conduction velocity; UE=upper extremity, LE= lower extremity; MCP=middle cerebellar peduncles; n.a.= not available.

**Figure 2:**
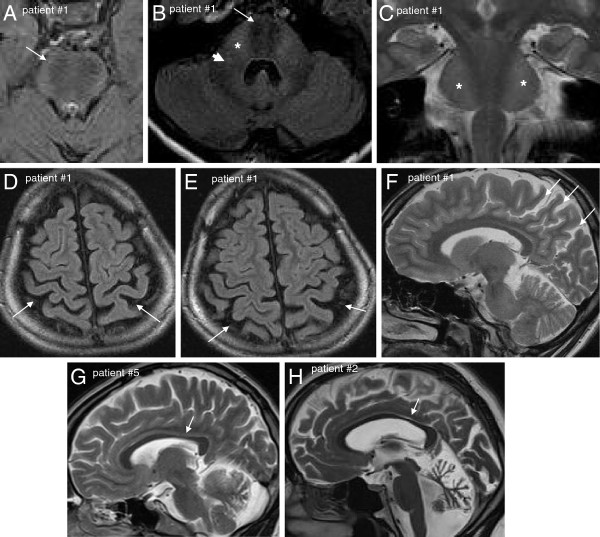
**Characteristic MRI findings in ARSACS.** Axial FLAIR images of upper pons (**A**) and middle pons (**B**) show hypointense strips in the central pons representing the cortico-spinal tract (white arrows): Moreover, on the level of the mid-pons (**B**), they reveal diffuse slight hyperintensity of the lateral pons when merging into the middle cerebellar peduncles (stars) and thickened middle cerebellar peduncles (white arrow head) in patient #1. Also on coronal T2 images (**C**) the bilateral hyperintensity of the lateral pons can be seen (stars). Bilateral post-central and parietal atrophy is shown for the same patient in axial FLAIR (**D, E**) and sagittal T2 (**F**) images (white arrows). Thinning of the posterior mid-body of the corpus callosum on sagittal T2 images is shown for patient #5 (**G**) and patient #2 (**H**) (white arrows).

**Figure 3:**
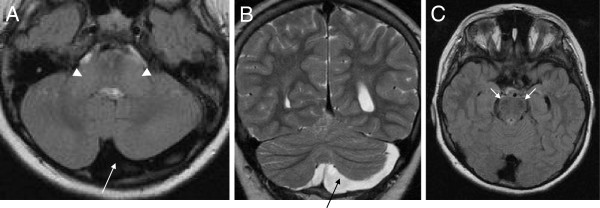
**Developmental asymmetry of cerebellar hemispheres.** Axial T1 (**A**) and coronal T2 (**B**) MRI images of patient #4 demonstrating developmental asymmetry of the cerebellar hemispheres (long arrows) without cerebellar atrophy. Apart from this finding, this patient shows the common infratentorial signs of ARSACS: thickening of the middle cerebellar peduncles (arrow head, **A**) and linear hypointensities of the mid-pons (best seen on axial FLAIR, arrows, **C**).

MRI (available from 8/9 patients) demonstrated cerebellar atrophy with predominance of the superior cerebellar vermis in 88% (7/8) (Table 3 and Figure 2). Interestingly, the single patient who was lacking cerebellar atrophy, did also not show any clinical signs of cerebellar dysfunction (but only mild afferent ataxia) (patient #4). His MRI did reveal, however, developmental asymmetry of the cerebellar hemispheres (Figure 3). All patients (8/8, 100%) showed linear pontine T2-*hypo*intensities, and, in addition, also bilateral FLAIR-T2-*hyper*intensities of the lateral pons when merging into the middle cerebellar peduncles (MCP) as well as thickening of the MCP (Figure 2). 75% (6/8) of the patients exhibited an arachnoidal cyst in the posterior fossa, 63% (5/8) bilateral parietal cerebral atrophy (Figure 2), and 63% (5/8) a short-stretched thinning of the posterior mid-body of the corpus callosum (Figure 2). While interrater reliability for rating of cerebellar atrophy (Cohens’s kappa: 1) and parietal atrophy (Cohen’s kappa: 0.8) was very good, it was only moderate for thinning of the posterior mid-body corpus callosum (Cohen’s kappa: 0.6). The lower interrater reliability with respect to the corpus callosum, however, does not lead to a possible over-reporting of corpus callosum abnormalities, but rather to a possible underestimation as in case of disagreement the more conservative estimate was adopted for final judgement.

### Phenotype of patients with two SACS variants of uncertain clinical significance class 3 (p.Thr458Ile and p. Val995Phe)

**Figure 4:**
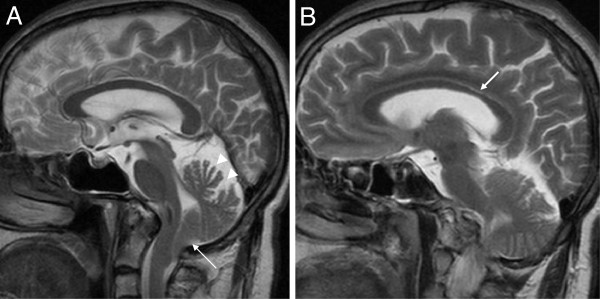
**Developmental posterior fossa malformation resembling Chiari type I in the Thr458Ile+ p.Thr458Ile patient.** (**A**) Midsagittal T2 MRI demonstrates herniation of cerebellar tonsils, resembling Chiari malformation type 1 (arrow). Also note the atrophy of the superior cerebellar vermis (arrow heads). (**B**) Para-midsagittal shows slight short-stretched thinning of the posterior mid-body of the corpus callosum (arrow).

Compared to the aforementioned ARSACS patients, the patient with the homozygous mutation p.Thr458Ile had a relatively late onset of ataxia at age 30 years, with absence of clinical and electrophysiological signs of pyramidal damage and neuropathy (no spasticity, no extensor plantar response, normal muscle tendon reflexes, normal vibration sense, normal nerve conduction studies, normal MEPs). Funduscopy revealed no increased demarcation of retinal nerve fibres. Like in other ARSACS patients, MRI revealed minor atrophy of the superior cerebellar vermis, an arachnoid cyst, and a thinning of the posterior mid-body of the corpus callosum (Figure 4). However, in contrast to all aforementioned ARSACS patients, no linear T2-hypointensities in the pons or T2-hyperintensities of the lateral pons and thickening of the MCP were observed. Interestingly, MRI revealed herniation of the cerebellar tonsils, resembling Chiari malformation type 1 (Figure 4). The patient being compound heterozygous for the p.Thr458Ile and p.Val995Phe variants showed an early-onset triad of cerebellar ataxia, pyramidal tract signs (bilateral spasticity and extensor plantar response) and peripheral sensorimotor neuropathy. However, nerve conduction studies revealed pure axonal damage without evidence for a demyelinating component, in contrast to all other ARSACS patients with neuropathy. Moreover, atrophy was observed not only of the cerebellar vermis and hemispheres (like in the other ARSACS patients), but also of the pons. Although thinning of the posterior mid-body of the corpus callosum was present, neither linear T2-hypointensities in the pons nor T2-hyperintensities of the lateral pons and thickening of the MCP could be detected.

## Discussion

In this study we identified 9 novel index patients with two pathogenic or likely pathogenic *SACS* variants. These patients were part of a consecutive series of n=172 patients with early-onset ataxia. Thus, ARSACS accounts for at least 5% (9/172) of early-onset ataxias - which makes it the second most common ataxia after Friedreich’s ataxia (29%; 50/172) in our series. *SACS* mutations were responsible for at least 11% (9/83) of patients with unexplained ataxia. This finding is comparable to recent studies observing relative frequencies of 12% (in 232 cerebellar ataxia patients [1]) and 13% (in 85 patients with at least 2 of the 3 cardinal features of ARSACS [5]).

The majority of our ARSACS patients (78%) presented with the classical early-onset triad of cerebellar ataxia, lower limb pyramidal tract signs and axonal-demyelinating sensorimotor peripheral neuropathy, thus corroborating other recent ARSACS case series [5,6,16]. However, one index patient and his likewise affected brother did not show cerebellar ataxia or clinical pyramidal tract signs (no spasticity, no extensor plantar response), and were thus initially presumed to suffer from Charcot-Marie-Tooth (CMT) Disease. This finding corroborates a recent finding of two ARSACS siblings presenting with a CMT-like picture [17]. Importantly, however, in contrast to these previously reported siblings, our index patient and his brother did not even show mild spasticity or cerebellar ataxia. This suggests that *SACS* should be considered in both complicated and uncomplicated CMT.

On the other side of the phenotypic spectrum – and in sharp contrast to these CMT-like subjects – neuropathy might also be absent in ARSACS as indicated by patient #8 who did not show clinical or electrophysiological evidence of peripheral neuropathy. So far, only one ARSACS individual has been reported without clinical and electrophysiologic signs of peripheral neuropathy, suffering from a very late onset phenotype (ataxia starting at age 40 years) [5]. Our finding demonstrates that even in early-onset cases, neuropathy is not an obligate feature of ARSACS. It might, however, develop at later stages of the disease.

Interestingly, patient #8 did also show neither spasticity nor extensor plantar response. So far, only two ARSACS individuals without lower-limb spasticity were described [4], but both displayed bilateral extensor plantar response, thus suggesting that pyramidal involvement might have been likely masked by the severe neuropathy in these two individuals [1]. Since neuropathy was absent in our index patient, the lack of spasticity cannot simply be explained by a masking neuropathy. Thus, taken together, in contrast to the name of this “spastic ataxia of Charlevoix-Saguenay”, neither spasticity (absent in 2/9=22%) nor extensor plantar response (absent in 3/9=33%) nor cerebellar ataxia (absent in 1/9=11%) are obligate features of this disease. Although pyramidal tract involvement is common in ARSACS patients, as illustrated by the fact that all of our patients had abnormal MEPs, it might be only subclinical in some of them.

Increased demarcation of retinal nerve fibres is also not an obligatory feature of ARSACS. It was exhibited only by 1 (17%) of our patients. This finding is line with other recent studies that observed this feature only 1 out of 8 [5] or in none out of 12 ARSACS patients [16], respectively. Taken together, these findings illustrate that increased demarcation of retinal nerve fibres might be a specific, but not obligatory finding in ARSACS [5], especially in non-Quebec ARSACS individuals [1]. While the high frequency of urge incontinence (77%) might be due to either autonomic dysfunction or pyramidal damage [18] (or a combination of both), erectile dysfunction observed in one out of three male patients aged ≥16 years (33%) is clearly an indicator of autonomic dysfunction. So far, erectile dysfunction has only been reported in one ARSACS patient [19], but might be underdiagnosed. Thus, patients with ARSACS should be actively investigated for autonomic disturbances.

Our imaging findings extend the range of diagnostic features that help to diagnose ARSACS in patients with unexplained ataxia, spasticity or CMT. All patients with two pathogenic *SACS* variants showed not only the well-established linear pontine T2-*hypo*intensities [2,3], but also bilateral FLAIR-T2-*hyper*intensities of the lateral pons as well as a thickening of the MCP. The structural correlate of this finding, which corroborates and extends recent findings from other groups [3,16,20], are probably abnormally large transverse pontocerebellar fibres [16]. The frequent finding of an arachnoidal cyst in the posterior fossa (75%) and of developmental cerebellar hemispheric atrophy in one patient underlines the hypothesis that disease processes in ARSACS are not only of neurodegenerative, but also of neurodevelopmental origin [16,18]. These findings might help to differentiate ARSACS from many other recessive neuropathic ataxias, since they are not found e.g. in Friedreich’s ataxia or POLG-associated ataxia [21].

It was recently suggested that structural brain damage in ARSACS is not limited to infratentorial regions [16]. Here we extend this notion, demonstrating for the first time that it also involves the cerebral cortex: 63% of our patients showed bilateral parietal cerebral atrophy. 63% of our patients also showed a short-stretched thinning of the posterior mid-body of the corpus callosum. Although this frequency is lower than in a previous study reporting a thin corpus callosum (TCC) in *all* patients [16], this finding substantiates the notion that disease processes in ARSACS also frequently involve key associative fibre bundles [16]. Thus, ARSACS should be part of the differential diagnosis in spastic ataxias with TCC, like e.g. SPG11, SPG15 [22] or *GBA2*[23].

We identified 17 novel *SACS* variants. Two of them – p.Thr458Ile and p.Val995Phe - were also observed in controls with a frequency of 0.4% or 0.3%, respectively. This indicates that they will likely be identified by many labs in the future, thus warranting a timely discussion whether they should be reported as pathogenic or not. The finding of a recessive mutation in a heterozygous state in controls does not *per se* refute a possible pathogenic role, in particular if the variant is common (as e.g. demonstrated by the expanded allele of *FXN* [Friedreich’s ataxia gene], which is observed with a heterozygous carrier rate of 1/60 to 1/90 in controls [24], or by the two frequent, clearly pathogenic c.1399 G>A [p.Ala467Thr] and c.2243 G>C [p.Trp748Ser] *POLG* mutations, which are observed with a heterozygous carrier rate of up to 1% in several European populations [25]). In particular, the p.Thr458Ile *SACS* variant might be pathogenic, given the *in silico* predictions, the high conservation index, and the MRI findings of a TCC as well as of a neurodevelopmental posterior fossa disorder consisting of an arachnoid cyst and herniation of tonsils in the homozygous carrier. Yet, for the time being, we here prefer not to claim any pathogenic significance, in particular given the large phenotypic differences compared to the other subjects with pathogenic *SACS* mutations. These findings illustrate a problem that is likely to occur frequently in the future: findings of variants with unknown significance may be frequent in *SACS* sequencing, given the fact that is has now entered clinical routine in many ataxia clinics. Thus, seemingly “atypical ARSACS phenotypes” should be scrutinized with particular caution and underlying *SACS* variants should ideally be analyzed functionally, e.g. with their impact on mitochondrial fission dynamics [26].

## Conclusions

In sum, we here demonstrate that each feature of the classical ARSACS triad (cerebellar ataxia, spasticity, peripheral neuropathy, Charlevoix-Saguenay origin) might in fact be missing in patients with *SACS* mutations, so the name might be misleading. Atypical presentations may be found much more frequently in the future when ARSACS genetics becomes a standard tool of ataxia work-up by new sequencing techniques and ataxia panel diagnostics. Nevertheless, characteristic MRI features – which also extend to supratentorial regions - will help to establish the diagnosis in most cases. Moreover, the electrophysiological finding of a demyelinating component of the sensorimotor peripheral neuropathy will help to distinguish ARSACS from other common recessive ataxias like Friedreich’s ataxia, *POLG* or AOA2, where neuropathy is almost exclusively of axonal type [21,27].

## Abbreviations

ARSACS: Autosomal recessive spastic ataxia Charlevoix-Saguenay; CMT: Charcot-Marie Tooth Disease; EVS: Exome variant server; FLAIR: Fluid attenuated inversion recovery; MCP: Middle cerebellar peduncles; MEP: Motor evoked potentials; MLPA: Multiplex Ligation-dependent Probe Amplification; MRI: Magnetic resonance imaging; NCS: Nerve conduction studies; POLG: Polymerase gamma; TCC: Thin corpus callosum; VUS: Variant of uncertain clinical significance

## Competing interests

Dr. Synofzik received a research grant by the Volkswagen Foundation, a travel grant by the Movement Disorders Society and AtaxiaUK/Ataxia Ireland, and consulting fees from Actelion Pharmaceuticals Ltd.

Dr. Soehn received a research grant by the International Parkinson Fonds (Deutschland) gGmbH (IPD).

Dr. Gburek-Augustat, Dr. Schicks, Dr. Karle, Dr. Schüle, Dr. Schöning, Dr. Biskup, Dr Rudnik-Schöneborn, Dr. Hoffmann, Dr. MacLeod, Dr. Schwarz, Dr. Krüger, and Dr. Kreuz report no disclosures.

Dr. Haack was supported by the NBIA disorders association.

Dr. Senderek received a grant by the Gebert Rüf Foundation.

Dr. Bender received a travel grant by Bayer Vital and was supported by research grants of the German Research Council (DFG) BE4609/1-1 and the Wilhelm-Schuler-Stiftung.

Dr. Peter Bauer received consulting honorary and speaker’s fees from Actelion Pharmaceuticals GmbH (Germany), Actelion Pharmaceuticals (Switzerland), and Centogene (Germany). He received research grants of the German Research Council (BMBF) to EUROSCAR.

Dr. Schöls received research grants of the Deutsche Forschungsgemeinschaft (SCHO754/4-1 and SCHO754/5-1), grants of the German Research Council (BMBF) to Leukonet (01GM0644) and mitoNET (01GM0864) and E-RARE grants to EUROSPA (01GM0807) and RISCA (01GM0820).

## Authors’ contributions

Dr. MS and Dr AS: design and conceptualization of the study, acquisition, analysis and interpretation of data; drafting the manuscript. Dr. JG-A, Dr. JS, Dr. KK, Dr. RS, Dr. TH, Dr. MS, Dr. SB, Dr SR-S, Dr. JS, Dr. K-TH, Dr, PM, Dr. JS, Dr. BB, Dr. SK, Dr. FK, Dr. PB: acquisition and analysis of data; revising the manuscript. Dr. LS: conceptualization of the study, acquisition, analysis and interpretation of data; revising the manuscript. All authors read and approved the final manuscript.

## Supplementary Material

Additional file 1 **A) *SACS* sequencing. B**) MLPA assay. **C**) Criteria for classification of sequence variants. Click here for file

Additional file 2 **Alignment results.** Multiple sequence alignments for novel *SACS* missense mutations identified in this study. Results were computed using the PolyPhen-2 interface for UCSC MultiZ46Way GRCh37/hg19 and the default Clustalx color coding: conserved A, C, F, H, I, L, M, V, W, Y or P residues are coloured blue, while conserved S or T residues are in green. Conserved positively charged residues and negatively charged residues are coloured red and magenta, respectively. Exact details can be found in the ClustalX manual (Thompson et al., 1997). Click here for file

Additional file 3 **Pedigree of an ARSACS patient with a second late-onset dominant cerebellar ataxia segregating in the pedigree.** The family history of index patient #2 (arrow) was positive for a three-generation late-onset (>30 years), slowly progressive, purely cerebellar autosomal-dominant ataxia. This led to a time- and cost-extensive work-up of dominant ataxia genes also in the index patient herself. After these dominant genes were all negative, also recessive genes were screened, leading to the identification of two pathogenic SACS variants (p. Arg728*; p.Phe4352Leufs*11), which were not observed in her affected relatives (WT= wild type). This observation illustrates that two different hereditary ataxias should be considered in pedigrees where the ataxia features differ strongly between pedigree members, e.g. early onset (in the index patient) vs. late onset (in her relatives), or multisystemic (in the index patient) vs. purely cerebellar (in her relatives). A timely consideration of this fact might help to find a correct diagnosis in a more timely manner and save resources. Click here for file

## References

[B1] Vermeer S van de Warrenburg BP Kamsteeg EJ Pagon RA, Bird TC, Dolan CR, Stephens K ARSACS GeneReviews [Internet] 2012 Seattle (WA): University of Washington http://www.ncbi.nlm.nih.gov/books/NBK1255/; accessed November 22nd, 2012

[B2] Gerwig M Kruger S Kreuz FR Kreis S Gizewski ER Timmann D Characteristic MRI and funduscopic findings help diagnose ARSACS outside Quebec Neurology 2010 75 2133 10.1212/WNL.0b013e318200d7f8 21135390

[B3] Gazulla J Benavente I Vela AC Marin MA Pablo LE Tessa A Barrena MR Santorelli FM Nesti C Modrego P New findings in the ataxia of Charlevoix-Saguenay J Neurol 2012 259 869 878 10.1007/s00415-011-6269-5 21993619

[B4] Shimazaki H Takiyama Y Sakoe K Ando Y Nakano I A phenotype without spasticity in sacsin-related ataxia Neurology 2005 64 2129 2131 10.1212/01.WNL.0000166031.91514.B3 15985586

[B5] Baets J Deconinck T Smets K Goossens D Van den Bergh P Dahan K Schmedding E Santens P Rasic VM Van Damme P Mutations in SACS cause atypical and late-onset forms of ARSACS Neurology 2010 75 1181 1188 10.1212/WNL.0b013e3181f4d86c 20876471

[B6] Vermeer S Meijer RP Pijl BJ Timmermans J Cruysberg JR Bos MM Schelhaas HJ van de Warrenburg BP Knoers NV Scheffer H Kremer B ARSACS in the Dutch population: a frequent cause of early-onset cerebellar ataxia Neurogenetics 2008 9 207 214 10.1007/s10048-008-0131-7 18465152 PMC2441586

[B7] Plon SE Eccles DM Easton D Foulkes WD Genuardi M Greenblatt MS Hogervorst FB Hoogerbrugge N Spurdle AB Tavtigian SV Sequence variant classification and reporting: recommendations for improving the interpretation of cancer susceptibility genetic test results Hum Mutat 2008 29 1282 1291 10.1002/humu.20880 18951446 PMC3075918

[B8] Zimprich A Benet-Pages A Struhal W Graf E Eck SH Offman MN Haubenberger D Spielberger S Schulte EC Lichtner P A mutation in VPS35, encoding a subunit of the retromer complex, causes late-onset Parkinson disease Am J Hum Genet 2011 89 168 175 10.1016/j.ajhg.2011.06.008 21763483 PMC3135812

[B9] Adzhubei IA Schmidt S Peshkin L Ramensky VE Gerasimova A Bork P Kondrashov AS Sunyaev SR A method and server for predicting damaging missense mutations Nat Methods 2010 7 248 249 10.1038/nmeth0410-248 20354512 PMC2855889

[B10] Kumar P Henikoff S Ng PC Predicting the effects of coding non-synonymous variants on protein function using the SIFT algorithm Nat Protoc 2009 4 1073 1081 10.1038/nprot.2009.86 19561590

[B11] Schwarz JM Rodelsperger C Schuelke M Seelow D MutationTaster evaluates disease-causing potential of sequence alterations Nat Methods 2010 7 575 576 10.1038/nmeth0810-575 20676075

[B12] Landis JR Koch GG The measurement of observer agreement for categorical data Biometrics 1977 33 159 174 10.2307/2529310 843571

[B13] Pollard KS Hubisz MJ Rosenbloom KR Siepel A Detection of nonneutral substitution rates on mammalian phylogenies Genome Res 2010 20 110 121 10.1101/gr.097857.109 19858363 PMC2798823

[B14] Grantham R Amino acid difference formula to help explain protein evolution Science 1974 185 862 864 10.1126/science.185.4154.862 4843792

[B15] Schmitz-Hubsch T du Montcel ST Baliko L Berciano J Boesch S Depondt C Giunti P Globas C Infante J Kang JS Kremer B Mariotti C Melegh B Pandolfo M Rakowicz M Ribai P Rola R Schols L Szymanski S van de Warrenburg BP Durr A Klockgether T Fancellu R Scale for the assessment and rating of ataxia: development of a new clinical scale Neurology 2006 66 1717 1720 10.1212/01.wnl.0000219042.60538.92 16769946

[B16] Prodi E Grisoli M Panzeri M Minati L Fattori F Erbetta A Uziel G D'Arrigo S Tessa A Ciano C Santorelli FM Savoiardo M Mariotti C Supratentorial and pontine MRI abnormalities characterize recessive spastic ataxia of Charlevoix-Saguenay. A comprehensive study of an Italian series Eur J Neurol Offic J Eur Federation Neurol Soc 2013 20 138 146 10.1111/j.1468-1331.2012.03815.x 22816526

[B17] Pyle A Griffin H Yu-Wai-Man P Duff J Eglon G Pickering-Brown S Santibanez-Korev M Horvath R Chinnery PF Prominent sensorimotor neuropathy due to SACS mutations revealed by whole-exome sequencing Arch Neurol 2012 69 1351 1354 22751902 10.1001/archneurol.2012.1472

[B18] Brodal P The Central Nervous System:Structure and Function: Structure and Function 2003 Oxford: Oxford University Press page 392

[B19] Miyatake S Miyake N Doi H Saitsu H Ogata K Kawai M Matsumoto N A novel SACS mutation in an atypical case with autosomal recessive spastic ataxia of Charlevoix-Saguenay (ARSACS) Intern Med 2012 51 2221 2226 10.2169/internalmedicine.51.7374 22892508

[B20] Gazulla J Vela AC Marin MA Pablo L Santorelli FM Benavente I Modrego P Tintore M Berciano J Is the ataxia of Charlevoix-Saguenay a developmental disease? Med Hypotheses 2011 77 347 352 10.1016/j.mehy.2011.05.011 21665375

[B21] Synofzik M Srulijes K Godau J Berg D Schols L Characterizing POLG ataxia: clinics, electrophysiology and imaging Cerebellum 2012 11 1002 1011 10.1007/s12311-012-0378-2 22528963

[B22] Schule R Schlipf N Synofzik M Klebe S Klimpe S Hehr U Winner B Lindig T Dotzer A Riess O Winkler J Schols L Bauer P Frequency and phenotype of SPG11 and SPG15 in complicated hereditary spastic paraplegia J Neurol Neurosurg Psychiatry 2009 80 1402 1404 10.1136/jnnp.2008.167528 19917823

[B23] Martin E Loss of Function of Glucocerebrosidase GBA2 Is Responsible for Motor Neuron Defects in Hereditary Spastic Paraplegia Am J Hum Genet 2013 92 238 244 10.1016/j.ajhg.2012.11.021 23332916 PMC3567271

[B24] Epplen C Epplen JT Frank G Miterski B Santos EJ Schols L Differential stability of the (GAA)n tract in the Friedreich ataxia (STM7) gene Hum Genet 1997 99 834 836 10.1007/s004390050458 9187683

[B25] Hakonen AH Davidzon G Salemi R Bindoff LA Van Goethem G Dimauro S Thorburn DR Suomalainen A Abundance of the POLG disease mutations in europe, australia, New zealand, and the united states explained by single ancient european founders Eur J Hum Genet 2007 15 779 783 10.1038/sj.ejhg.5201831 17426723

[B26] Girard M Lariviere R Parfitt DA Deane EC Gaudet R Nossova N Blondeau F Prenosil G Vermeulen EG Duchen MR Mitochondrial dysfunction and Purkinje cell loss in autosomal recessive spastic ataxia of Charlevoix-Saguenay (ARSACS) Proc Natl Acad Sci USA 2012 109 1661 1666 10.1073/pnas.1113166109 22307627 PMC3277168

[B27] Anheim M Monga B Fleury M Charles P Barbot C Salih M Delaunoy JP Fritsch M Arning L Synofzik M Ataxia with oculomotor apraxia type 2: clinical, biological and genotype/phenotype correlation study of a cohort of 90 patients Brain 2009 132 2688 2698 10.1093/brain/awp211 19696032

