# Supplementary material for: Autosomal recessive spastic ataxia of Charlevoix Saguenay (ARSACS): expanding the genetic, clinical and imaging spectrum
Source: Orphanet J Rare Dis. 2013 Mar 15;8:41. doi: 10.1186/1750-1172-8-41 (PMC3610264; doi:10.1186/1750-1172-8-41)
Supplement: Additional file 1 — A) SACS sequencing. B) MLPA assay. C) Criteria for classification of sequence variants. [file 1750-1172-8-41-S1.doc]

**Supplement 1:**

**A) *SACS* sequencing**

Accession number: ENST00000382298 (Ensembl), NM_014363.4 (NCBI)

Genomic coordinates (GRCh37): 23,902,965 - 24,007,841

Cytogenetic location: 13q12.12

**Table S1 Primer design for *SACS* sequence analysis**

SACS coding exons and flanking intronic sequences were amplified using the primer pairs given in Table S1. Primers were 5' tailed with M13 universal sequences to allow sequencing with M13 universal primers (M13-fw: 5’-gtaaacgacggccagtg-3’; M13-rev: 5’-ggaaacagctatgaccatga-3’).

Exon 8 had to be amplified in a target-specific long range PCR followed by nested PCR amplifications due to a homologous region on chromosome 11.

| **Exon** | **Forward (5’ → 3’)** | **Reverse (5’ → 3’)** | **Fragment length (including M13)** |
| --- | --- | --- | --- |
| 2 | GCAGGAAGAGCTGAACTTGG | CAACAAGTCTTAAAATCCCAATG | 232 |
| 3 | GATGTGGGAGAGGTAACGC | CAACTCAGGACAAGCACAGG | 425 |
| 4 | CCGTGGAATATTCACTTCTCC | GTGGTGAGCTGAGATTGTGC | 422 |
| 5 | CATTGGTGACAATTCATGGG | TACACTGCAGCCACAGAGG | 329 |
| 6 | TGTTGCAAATAGTGGGTTTCC | TGATAGACTGTTCATTAAACAAAGC | 367 |
| 7 | GAGATAGAACAGAACACCCTGG | TGGCTTTTAAACAGTATCACCTC | 346 |
| 8 long range | TGGATTGAATCAAAAGAGAAATC | TCACAGTGAGCAGGAGCAG |  |
| 8-1 nested | TTTAATGGCTCATGCTTTTTC | GAAGGTTGTAGGCGAAGAGG | 369 |
| 8-2 nested | TGTTGGCATTTTTGGAAGC | GGACAAATTTCAGTTCATCAGC | 540 |
| 8-3 nested | GACTCCAAGCAATAACATCACC | CAGTGCACCTTGACTTTGC | 586 |
| 8-4 nested | ATGAATGTTGTCCCCAAAGC | CCCAAGCAGCTCACTGTAGG | 541 |
| 8-5 nested | GTACCAGGGAATGTGGATGC | TCACAGTGAGCAGGAGCAG | 383 |
| 9 | TTTAAAATCTTGGAATTGTGGG | AGCTTGAGCCATAAGAAATTG | 302 |
| 10-1 | TTTGTGAGAATAGAAAGCTGTTG | ATCTGCAAAACAGCACTTGG | 557 |
| 10-2 | TGTGGAACTCATTAGACTCAGG | GAGAAGATAGATTCTCAAGGACCC | 663 |
| 10-3 | AGATCTGCGACTTTCTATTTCAG | GCTGGAACCCATTTTATTTTC | 640 |
| 10-4 | AAAGGATGTTGTGCAAGTGG | ACCCATGGAAATTTTAAGGC | 571 |
| 10-5 | TCTTCAAAAACCTTTAGTGATGAAG | TCTTCAAGTAAGTCATTAAGGTCATC | 573 |
| 10-6 | CCCAACACACCAGTTCCTATAC | CAACTTTGTCAACTTCTCCCC | 575 |
| 10-7 | CTCCTAGACCCAGGGATGG | CCACAGAGACTAAATTCATCCAC | 552 |
| 10-8 | TAATGGAACCCTTTTCCGAC | TCCATGCAAGTACACAGAAGC | 595 |
| 10-9 | ATTGCTGATTTACAGTCGCC | CCTTGGCAAATTACAGAAAAATC | 603 |
| 10-10 | AACAGATACAAAAGGACGATGG | TTAAAGGATCTCTAAGTTCTGCTTC | 564 |
| 10-11 | GGATTTGAAGAAGCTGGCTG | AGCAAAATCCTTTGCTCTAGG | 553 |
| 10-12 | AGAACGTGCAGTGTCAGTAGC | TTTTCTGAGTCAACATATGCATTC | 619 |
| 10-13 | GAAGCCAACAGTTGATCTGG | GGCAATCATTGTAGCATAACG | 594 |
| 10-14 | CGAATAATCAGTGAAGGAATATGG | CAAGATTCTGAATTCCTCTAACATC | 584 |
| 10-15 | TTGATCCTAGACAGCATCCAG | TCCATGTGATTAAGAAACATTAGAAG | 578 |
| 10-16 | TTGCACAATGTTCAGATTTCC | AAATGGCAGCCCAGTCTC | 599 |
| 10-17 | CGTGGCTAATTTGTAATAGATCAGG | AGCCATCAATATTTGGAGCC | 598 |
| 10-18 | TTTTCCCAGTTAACCGTCTTG | ACTTCAATCTCATTTTCTTCTGC | 602 |
| 10-19 | TTTTTAATGACATTTTCCTCTCC | AAGCAATGCCCAGTCTTTTAG | 579 |
| 10-20 | AAAAGTTGCACAAAGTGGAAAG | GGATTTATAGCACGGAAGTGAC | 598 |
| 10-21 | CACAAGCATCTTGAAGGCTC | TTCAGGAAGCATAACTTCAAAAAC | 626 |
| 10-22 | GCTGAGGAATTATCAGAGATTAAGG | CCTCTTGATATTGAGGATGAAATC | 536 |
| 10-23 | CAAAATACAGTTGATATCCTTCTGC | GGCTTCAGAAGTTTCCAACC | 597 |
| 10-24 | GATGAAGAAATGGTAAAAACTAGAGC | AACATTTGCACACCAATATTCC | 582 |
| 10-25 | AAGCCAGGATGGTAGATTGG | AGTTTCACTTCTGCTGTGGG | 524 |
| 10-26 | TAATGCTTTTCTGGCCAATG | TGATCCATAGATATCACCACCTTC | 554 |
| 10-27 | CCTGCTGAAATTCATTACACTC | CCATTTCAAATACAACCGCC | 565 |
| 10-28 | TCTTCCAAACATCAGTCCCC | TAGTCAGCTGCAATCAAAGC | 642 |
| 10-29 | TCAGCCGGACAGACTTACTC | GAATTCTCCAAGAACAATCTGC | 641 |

**B) MLPA assay**

**Table S2 MLPA probe design for *SACS* gene dosage analyses**

The table lists the hybridising sequences and optional stuffer sequences for Left Probe Oligonucleotides (LPO) and Right Probe Oligonucleotides (RPO). Each LPO contains an additional binding sequence of a forward PCR primer (5’-GGGTTCCCTAAGGGTTGGA-3’) at its 5’ end, and similarly each RPO ends with a binding sequence for a reverse PCR primer (5’-TCTAGATTGGATCTTGCTGGCAC-3’) at its 3’ end.

MLPA probes were added to the SALSA MLPA P300 Human reference probemix (MRC Holland) in the indicated combinations giving a total of two probemixes to enable the usage of similar probe length.

|  | **Exon** | **Left hybridising sequence (LHS) +** *optional* *stuffer*  **(5’ → 3’)** | **Right hybridising sequence (RHS) +** *optional stuffer* **(5’ → 3’)** | **Length LPO** | **Length RPO** | **Total probe length** |
| --- | --- | --- | --- | --- | --- | --- |
|  |  |  |  |  |  |  |
| **Mix 1** | 2 | *t*AGGATCACAGAAGTGAGGCCAAGAT | GCCCATGTCACCAGAGCCAGAAGAAA*acc* | 45 | 52 | 97 |
|  | 6 | *tctgaaagaa*CAATACGGAACAGAGACTCTTTGGTCAAA | AGATATGGCGCCATATCAGGGTAAGAATC*gatgg* | 58 | 57 | 115 |
|  | 7 | *t*CCAGAGGACTGGCACGGCATTCAAGAAATAG | CAAGAAGCAGGAAAAAGGATGATCCTCTGAA | 51 | 54 | 105 |
|  | 9 | *caggcgctg*CAGGTCCCTTTTCCCAAGTCTTGAAGGAAGATTTATTTT | GGATAACTTGAAACCTCACCTTGTGGCTGCTTTAAAGGA*tttcc* | 67 | 67 | 134 |
|  | 10-2 | *gtcatgtca*CAGATCACAGTGGAAGAATTTCACCATGTG | TTCAGAAGGATTGCTGATTTACAGTCGCCA*gagcagaaa* | 58 | 62 | 120 |
|  | 10-4 | ctttacgcccgttttctggCCCACTTTCAAGTCGGTTGGCAATCCAGTGGAA | GCACGCAGATGGCTAAGACAAGCCAGAGCAAACT*atgcggtgaacttc* | 71 | 71 | 142 |
|  |  |  |  |  |  |  |
| **Mix 2** | 3 | tatgtggcCGACTGAGCGTCCCTCTGCTT | TCCACAGGTGGGTCCCGGTGA*tcggtg* | 48 | 50 | 98 |
|  | 4 | cacgcccaccatCCAAAAATTGTCATCTTTTTGTAAACCTTCAATCAAAAG | GCTTAAAAGGGGGAGGTATAATATTGACTTTACCAGAAG*tcccaccct* | 70 | 71 | 141 |
|  | 5 | gactccacggtTGAGAAGATATCCAGAAGGAGGACAGATTC | TTAAGGTGGGGCACATGTCGTGTTCATGTG*agatgagc* | 60 | 61 | 121 |
|  | 8 | gactggcaGTGCTGCTCTTTCTGAAAAGTGTGCAGGAT | GTTTCCTTATATGTCCGAGAGGCTGACGGA*gctga* | 57 | 58 | 115 |
|  | 10-1 | gaggagaaatataCAGACATGTGTGGAACTCATTAGACTCAGGATTCC | ATCGTTAGTCATTTTAGACGATGAATCTGAAGCAC*aactgaacaa* | 67 | 68 | 135 |
|  | 10-3 | ttCAACTATAAAAAACCCCATAGGGCCTTCTGT | TTTTTGCCTCTTTCTTTGGAGACTGGGCTGC | 52 | 54 | 106 |

**C) Criteria for classification of sequence variants**

**Table S3 Classification of sequence variants**

Sequence variants were classified using a five-class-system as proposed by Plon et al. 2008 for the reporting of cancer susceptibility genetic variants. Importantly, this classification system distinguishes three classes of variants of uncertain clinical significance (VUS) in order to differentiate variants for which there is too little

information to make an assessment (class 3) from those for which there is significant, but not definite, evidence against (class 2) or for (class 4) pathogenicity. The clinical significance of a particular variant was assessed based on occurrence and frequency in the general population (dbSNP database, exome variant server, population controls), reports in the literature and mutation databases (http://www.medgen.mcgill.ca/SACSIN), sequence conservation (as mirrored by phyloP index), physico-chemical differences of involved amino acids and effects on protein structure, location in known protein domains, *in-silico* predictions (PolyPhen-2, SIFT, MutationTaster), or functional assay results, co-occurrence in our cohort, and cosegregation in the family of an index patient.

| **Class** | **Type** | **Description** |
| --- | --- | --- |
| 1 | neutral | Not Pathogenic or of No Clinical Significance |
| 2 | VUS | Likely Not Pathogenic or of Little Clinical Significance |
| 3 | VUS | Uncertain |
| 4 | VUS | Likely Pathogenic |
| 5 | deleterious | Definitely Pathogenic |

Reference:

[Plon SE](http://www.ncbi.nlm.nih.gov/pubmed?term=Plon SE%5BAuthor%5D&cauthor=true&cauthor_uid=18951446), [Eccles DM](http://www.ncbi.nlm.nih.gov/pubmed?term=Eccles DM%5BAuthor%5D&cauthor=true&cauthor_uid=18951446), [Easton D](http://www.ncbi.nlm.nih.gov/pubmed?term=Easton D%5BAuthor%5D&cauthor=true&cauthor_uid=18951446), [Foulkes WD](http://www.ncbi.nlm.nih.gov/pubmed?term=Foulkes WD%5BAuthor%5D&cauthor=true&cauthor_uid=18951446), [Genuardi M](http://www.ncbi.nlm.nih.gov/pubmed?term=Genuardi M%5BAuthor%5D&cauthor=true&cauthor_uid=18951446), [Greenblatt MS](http://www.ncbi.nlm.nih.gov/pubmed?term=Greenblatt MS%5BAuthor%5D&cauthor=true&cauthor_uid=18951446), [Hogervorst FB](http://www.ncbi.nlm.nih.gov/pubmed?term=Hogervorst FB%5BAuthor%5D&cauthor=true&cauthor_uid=18951446), [Hoogerbrugge N](http://www.ncbi.nlm.nih.gov/pubmed?term=Hoogerbrugge N%5BAuthor%5D&cauthor=true&cauthor_uid=18951446), [Spurdle AB](http://www.ncbi.nlm.nih.gov/pubmed?term=Spurdle AB%5BAuthor%5D&cauthor=true&cauthor_uid=18951446), [Tavtigian SV](http://www.ncbi.nlm.nih.gov/pubmed?term=Tavtigian SV%5BAuthor%5D&cauthor=true&cauthor_uid=18951446); [IARC Unclassified Genetic Variants Working Group](http://www.ncbi.nlm.nih.gov/pubmed?term=IARC Unclassified Genetic Variants Working Group%5BCorporate Author%5D).

Sequence variant classification and reporting: recommendations for improving the interpretation of cancer susceptibility genetic test results.

[Hum Mutat.](http://www.ncbi.nlm.nih.gov/pubmed/18951446) 2008 Nov;29(11):1282-91. doi: 10.1002/humu.20880.
