# Supplementary material for: Autosomal recessive spastic ataxia of Charlevoix Saguenay (ARSACS): expanding the genetic, clinical and imaging spectrum
Source: Orphanet J Rare Dis. 2013 Mar 15;8:41. doi: 10.1186/1750-1172-8-41 (PMC3610264; doi:10.1186/1750-1172-8-41)
Supplement: Additional file 2 — Alignment results. Multiple sequence alignments for novel SACS missense mutations identified in this study. Results were computed using the PolyPhen-2 interface for UCSC MultiZ46Way GRCh37/hg19 and the default Clustalx color coding: conserved A, C, F, H, I, L, M, V, W, Y or P residues are coloured blue, while conserved S or T residues are in green. Conserved positively charged residues and negatively charged residues are coloured red and magenta, respectively. Exact details can be found in the ClustalX manual (Thompson et al., 1997). [file 1750-1172-8-41-S2.doc]

**Supplement 2**


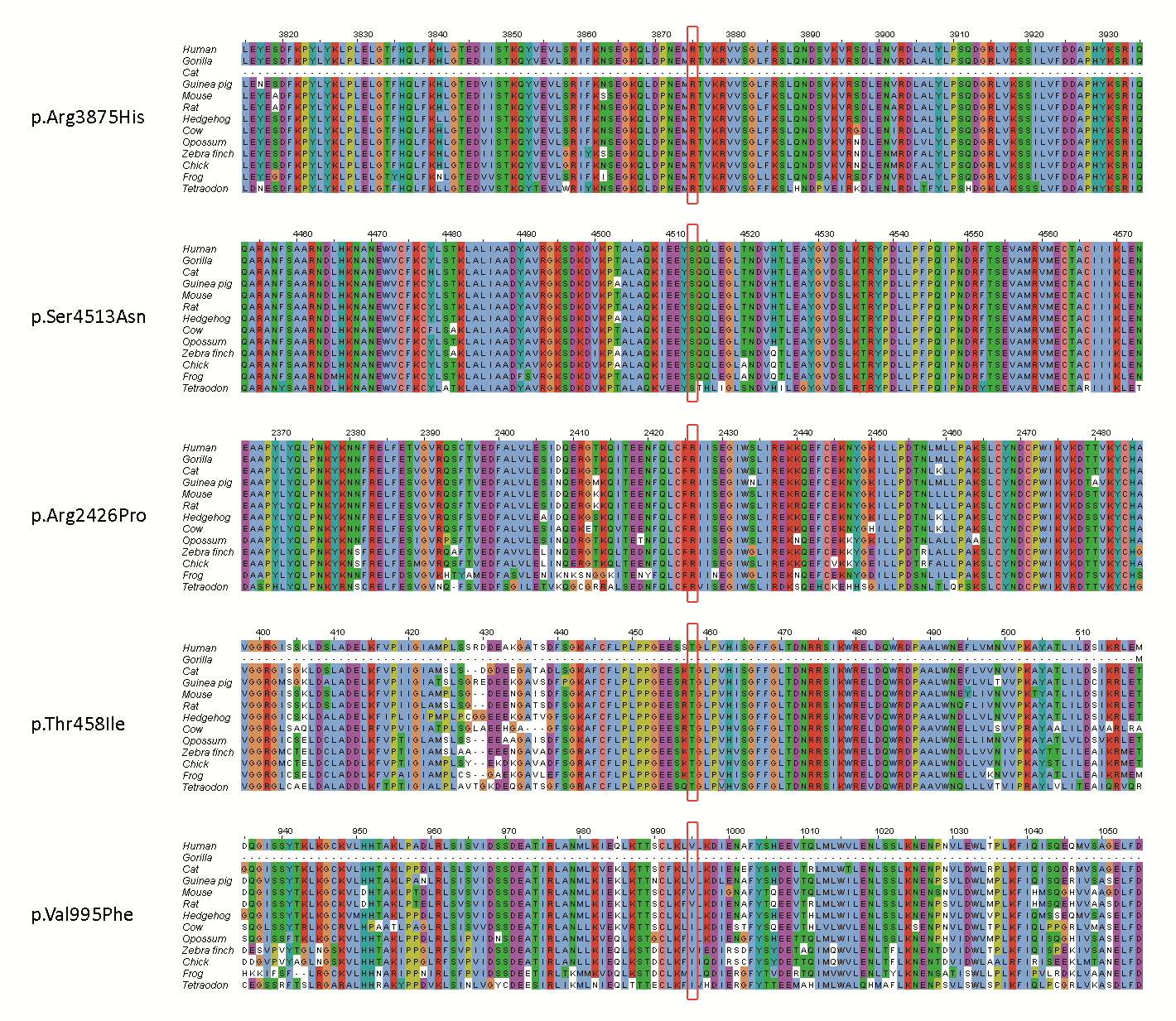


**Supplement 2: Alignment results.** Multiple sequence alignments for novel *SACS* missense mutations identified in this study. Results were computed using the PolyPhen-2 interface for UCSC MultiZ46Way GRCh37/hg19 and the default Clustalx color coding: conserved A, C, F, H, I, L, M, V, W, Y or P residues are coloured blue, while conserved S or T residues are in green. Conserved positively charged residues and negatively charged residues are coloured red and magenta, respectively. Exact details can be found in the ClustalX manual (Thompson et al., 1997).

**References:**

Thompson, J. D., Gibson, T. J., Plewniak, F., Jeanmougin, F. & Higgins, D. G. (1997). The CLUSTAL_X Windows interface: flexible strategies for multiple sequence alignment aided by quality analysis tools. Nucleic Acids Res 25, 4876-4882.
