# Supplementary material for: Autosomal recessive spastic ataxia of Charlevoix Saguenay (ARSACS): expanding the genetic, clinical and imaging spectrum
Source: Orphanet J Rare Dis. 2013 Mar 15;8:41. doi: 10.1186/1750-1172-8-41 (PMC3610264; doi:10.1186/1750-1172-8-41)
Supplement: Additional file 3 — Pedigree of an ARSACS patient with a second late-onset dominant cerebellar ataxia segregating in the pedigree. The family history of index patient #2 (arrow) was positive for a three-generation late-onset (>30 years), slowly progressive, purely cerebellar autosomal-dominant ataxia. This led to a time- and cost-extensive work-up of dominant ataxia genes also in the index patient herself. After these dominant genes were all negative, also recessive genes were screened, leading to the identification of two pathogenic SACS variants (p. Arg728*; p.Phe4352Leufs*11), which were not observed in her affected relatives (WT= wild type). This observation illustrates that two different hereditary ataxias should be considered in pedigrees where the ataxia features differ strongly between pedigree members, e.g. early onset (in the index patient) vs. late onset (in her relatives), or multisystemic (in the index patient) vs. purely cerebellar (in her relatives). A timely consideration of this fact might help to find a correct diagnosis in a more timely manner and save resources. [file 1750-1172-8-41-S3.doc]

**Supplement 3**

*38 years

AoO: 6 years

progression: fast

negative for: SCA 1,2,3,6,7,8,

SCA 10,12,14,17, 27, DRPLA,

***SACS:* p.[Arg728*] ;[Phe4352Leufs*11]**

*80yrs

02.05.1932

*83yrs

cerebellar ataxia

impaired upward gaze

AoO: 62yrs

progression: slow

***SACS*: WT**

*81yrs

*80yrs

† 70yrs

„speech and balance

difficulties“

*58 yrs

cerebellar ataxia

reduced tendon reflexes

AoO: 30‘s

negative for SCA1,2,6,7,8

***SACS*: WT**

*82 yrs

**Supplement 3: Pedigree of an ARSACS patient with a second late-onset dominant cerebellar ataxia segregating in the pedigree.**

The family history of index patient #2 (arrow) was positive for a three-generation late-onset (>30 years), slowly progressive, purely cerebellar autosomal-dominant ataxia. This led to a time- and cost-extensive work-up of dominant ataxia genes also in the index patient herself. After these dominant genes were all negative, also recessive genes were screened, leading to the identification of two pathogenic *SACS* variants (p. Arg728*; p.Phe4352Leufs*11), which were not observed in her affected relatives (WT= wild type).

This observation illustrates that two different hereditary ataxias should be considered in pedigrees where the ataxia features differ strongly between pedigree members, e.g. early onset (in the index patient) vs. late onset (in her relatives), or multisystemic (in the index patient) vs. purely cerebellar (in her relatives). A timely consideration of this fact might help to find a correct diagnosis in a more timely manner and save resources.
